# Integrative Bayesian tensor regression for imaging genetics applications

**DOI:** 10.3389/fnins.2023.1212218

**Published:** 2023-08-23

**Authors:** Yajie Liu, Nilanjana Chakraborty, Zhaohui S. Qin, Suprateek Kundu, The Alzheimer’s Disease Neuroimaging Initiative

**Affiliations:** ^1^Department of Biostatistics and Data Science, School of Public Health, The University of Texas Health Science Center at Houston, Houston, TX, United States; ^2^Department of Biostatistics, Epidemiology and Informatics, Perelman School of Medicine, University of Pennsylvania, Philadelphia, PA, United States; ^3^Department of Biostatistics and Bioinformatics, Rollins School of Public Health, Emory University, Atlanta, GA, United States; ^4^Department of Biostatistics, Division of Basic Science Research, The University of Texas MD Anderson Cancer Center, Houston, TX, United States

**Keywords:** Alzheimer's disease, Bayesian tensor regression models, collinearity, imaging genetics analysis, transcriptomics

## Abstract

Identifying biomarkers for Alzheimer's disease with a goal of early detection is a fundamental problem in clinical research. Both medical imaging and genetics have contributed informative biomarkers in literature. To further improve the performance, recently, there is an increasing interest in developing analytic approaches that combine data across modalities such as imaging and genetics. However, there are limited methods in literature that are able to systematically combine high-dimensional voxel-level imaging and genetic data for accurate prediction of clinical outcomes of interest. Existing prediction models that integrate imaging and genetic features often use region level imaging summaries, and they typically do not consider the spatial configurations of the voxels in the image or incorporate the dependence between genes that may compromise prediction ability. We propose a novel integrative Bayesian scalar-on-image regression model for predicting cognitive outcomes based on high-dimensional spatially distributed voxel-level imaging data, along with correlated transcriptomic features. We account for the spatial dependencies in the imaging voxels via a tensor approach that also enables massive dimension reduction to address the curse of dimensionality, and models the dependencies between the transcriptomic features via a Graph-Laplacian prior. We implement this approach via an efficient Markov chain Monte Carlo (MCMC) computation strategy. We apply the proposed method to the analysis of longitudinal ADNI data for predicting cognitive scores at different visits by integrating voxel-level cortical thickness measurements derived from T1w-MRI scans and transcriptomics data. We illustrate that the proposed imaging transcriptomics approach has significant improvements in prediction compared to prediction using a subset of features from only one modality (imaging or genetics), as well as when using imaging and transcriptomics features but ignoring the inherent dependencies between the features. Our analysis is one of the first to conclusively demonstrate the advantages of prediction based on combining voxel-level cortical thickness measurements along with transcriptomics features, while accounting for inherent structural information.

## 1. Introduction

Alzheimer's disease (AD) is a significant public health concern, affecting millions of people worldwide (Association et al., 2014). The disease's prevalence is expected to rise in the coming decades due to the aging of the population (Brookmeyer et al., 2007). There is currently no gold standard cure for Alzheimer's Disease, but the use of biomarkers such as neuroimaging and -omics variables have been shown to improve the accuracy of diagnosing Alzheimer's disease, particularly in its early stages. This is important because early detection can lead to earlier treatment and better outcomes for patients. Statistical and machine learning methods for discovering biomarkers have relied on validating their success in terms of either classifying the disease status (AD vs non-AD and so on) or predicting cognitive outcomes that are known to deteriorate with the progression of AD.

Imaging biomarkers play an increasingly important role in the diagnosis of AD and mild cognitive impairment (MCI). Magnetic resonance imaging (MRI) examination is a standard clinical assessment of patients with dementia. It has been shown that there is high correlation between brain atrophy deduced from structural MRI and AD progression (Frisoni et al., 2010). A highly sensitive imaging biomarker for AD representing structural atrophy is the cortical thickness (CT) (Du et al., 2007; Weston et al., 2016). In this context, MRI measurements of cortical thinning may prove to be better distinguishing markers than volumetric measurements (Du et al., 2007). Genetic heterogeneity between cortical measures and brain regions have been established in cognitive normal individuals (Sabuncu et al., 2012; Hofer et al., 2020), and it has been found that pathways involved in the cellular processes and neuronal differentiation may lead to neuronal loss, cortical thinning and AD (Kim et al., 2020). Aging related cortical thinning may be linked to genetic effects on regional variations in cortical thickness in middle age (Fjell et al., 2015), and longitudinal CT changes in the hippocampus region may be due to the combined effect of multiple genetic risk factors (Harrison et al., 2016). High resolution brain images that capture the cortical thickness across different voxels or regions can therefore serve as crucial variables of interest in the study of the progression of neurodegenerative diseases and their association with genetics and transcriptomics.

In addition to imaging biomarkers, there has been a parallel interest in discovering genetic signatures driving AD progression. Most genetic association studies are based on case-control designs, and as such they rely on a crude indicator of disease status. Unfortunately, this approach has not been overly successful in terms of identifying reproducible genetic signals, despite many studies suggesting potential susceptible loci. Existing genome-wide association studies that are primarily based on sporadic AD have identified over 50 loci associated with AD, but many potentially important genetic factors driving AD remains to be discovered (Bellenguez et al., 2020; Sims et al., 2020). Another branch of literature has examined the association between cognitive abilities and genetic factors (McGue et al., 1993; Plomin and Spinath, 2002). The overwhelming majority of existing genetic studies on AD have focused on single nucleotide polymorphisms (SNPs). However, emerging studies revealed that alternative gene expression regulation mechanisms, such as mRNA-transcription factor interactions, or copy number variants, could also impact neurodegeneration (Annese et al., 2018). Readers can refer to Bagyinszky et al. (2020) for a review.

Given that AD is a complex disease whose progression is affected by biological changes at multiple levels, there has been an increasing (and relatively recent) focus on integrative approaches that combine multiple types of features at different scales. Along these lines, several studies have adopted a multimodal approach to AD classification and cognitive prediction, which have included multiple types of imaging data such as structural (MRI) and functional (PET) imaging (Hao et al., 2020; Dartora et al., 2022) along with CSF biomarkers (Tong et al., 2017). Furthermore, brain imaging genomics, which is a term for integrated analysis of brain imaging and genomics data, along with other clinical and environmental data is gaining rapid popularity in different mental disorders (Shen and Thompson, 2019), and particularly in AD studies (Nathoo et al., 2019). Most brain imaging genomics approaches for predicting AD have relied on structural imaging and SNP data (Zhang et al., 2011; Kong et al., 2015; Dukart et al., 2016; Li et al., 2022). Often low rank models are used for integrating imaging and SNP data for prediction as in Kong et al. (2020), or two-step approaches are used to tackle the high-dimensional features as in Yu et al. (2022) who proposed a causal analysis method to map the Genetic-Imaging-Clinical pathway for Alzheimer's Disease. Other approaches for fusing functional imaging (resting state fMRI) and genetic features for classifying AD disease classes have also been proposed (Bi et al., 2021).

Although the above multimodal approaches and related methods that combine imaging and genetics features for modeling AD outcomes are useful, they are unfortunately beset with one or more pitfalls. First, existing imaging genetics methods may not account for the spatial configurations of imaging voxels as well as the inherent dependencies of the genetic features, which may lead to potential collinearity issues and loss of power and prediction accuracy. Some methods use dimension reduction such as principal component analysis or canonical correlation analysis to reduce the dimension of the gene features and reduce collinearity. However, these and related data fusion methods for data integration to create lower dimensional features may lead to a loss of interpretability and possible information loss. Second, most methods use region-level brain imaging features that smooth over voxel-level information acquired from the image, which is possibly done to reduce the curse of dimensionality. However, region-level analysis potentially results in less granular interpretations and loss of information that in turn can affect prediction/classification performance. Further, such aggregation under a region-level analysis may not be suitable for sparse cortical thickness measurements as elaborated in the sequel. Thirdly, the overwhelming majority of the limited literature on prediction/classification methods based on imaging and genetics features rely on frequentist approaches that report point estimates and do not capture uncertainty that is highly desirable for high-dimensional imaging applications involving noisy images. Last but not the least, most integrative imaging genetics approaches use SNPs data, but there are limited approaches that are specifically designed to combine imaging and transcriptomics data to our knowledge. There are several reasons that we believe the transcriptomics data offer advantages over genetics features (variants). First, the transcriptomics data combines both genetics information along with environmental influences. Second, the number of genes is much smaller than the number of mutations (mostly single nucleotide polymorphisms), by several orders of magnitude, which is expected to ease potential difficulties with curse of dimensionality associated with SNP based analysis. As a result, the burden on multiple comparison adjustment is greatly alleviated. Third, it is cheaper and easier to obtain transcriptomics data than genotyping data in a clinical setting.

In this article, we propose a novel approach to predict cognitive outcomes in AD by integrating voxel-level cortical thickness measurements derived from T1w-MRI along with transcriptomics (gene expression) features. We propose a novel Bayesian structured regression approach that accounts for the spatial orientation of the voxels in the brain image via a tensor representation for the imaging coefficients, and simultaneously accounts for dependency between genetic features via a graph Laplacian structure. We illustrate that the use of a tensor-based approach can provide valuable insights into the structure and organization of complex data such as brain images. By taking into account the spatial relationships between voxels, it is possible to uncover patterns and relationships that may be missed by routinely used voxel-wise analysis. From a methodological standpoint, the proposed approach can be considered as an extension of the scalar-on-tensor regression approach in Guhaniyogi et al. (2017) to the case of sparse images (representing cortical thickness measurements in our context) and to include high-dimensional and collinear genetic features. The latter is made possible based on the adoption of the approach proposed in Liu et al. (2014). Although there are alternative methods to incorporate dependency between genes, such as via gene networks (Chang et al., 2018), the graph Laplacian structure provides a more flexible strategy to incorporate dependence that is broadly applicable to different types of -omics features such as SNPs or discrete copy number variations, and does not run the risk of producing inaccurate results when the graph knowledge is misspecified. We implement an efficient Markov chain Monte Carlo (MCMC) strategy that draws samples from the posterior distribution, and estimated parameters via the posterior mean.

From the application perspective, we use the proposed approach to predict cognitive scores in Alzheimer's Disease Neuroimaging Initiative (ADNI) data. Our analysis has several novelties and is distinct from existing methods for whole brain genomics prediction analysis in literature. First, unlike existing methods that typically use SNP features, we use transcriptomics features and account for the unknown dependencies between these features. Second, we use high-dimensional voxel-level cortical thickness features instead of routinely used region-level measurements. This is indeed critical for our analysis since cortical thickness is only measured on a sparse set of voxels in the brain depending on the configuration of the brain cortex, and a region level analysis that averages over all voxels within pre-defined region of interest (ROI) may not provide sensible results. This is due to the fact that each ROI is expected to average over a non-ignorable proportion of voxels with zero cortical thickness that will potentially render the ROI level cortical thickness measurements as unreliable. Instead, our analysis at the voxel-level explicitly accounts for voxels with zero cortical thickness without any information loss resulting from ROI level averaging. Third, another innovation is that we analyse longitudinal cognitive scores using imaging data from baseline, and months 6 and 12 follow-up along with cross-sectional transcriptomic data. Performing the analysis at three longitudinal time points enables us to validate a set of robust transcriptomics features that show consistent signals across multiple visits, and therefore are potentially more reliable and reproducible. In addition, we perform another set of novel analysis that involves the prediction of the change in cognitive scores between visits based on the change in the voxel-wise cortical thickness maps across visits, along with transcriptomics and demographic features. The goal of this second analysis is to investigate the ability of the longitudinally varying imaging features and the transcriptome measurements to predict cognitive changes over time. Rigorous comparisons illustrate considerable improvements in prediction performance of the proposed integrative brain imaging transcriptomics approach compared to a similar analysis that just uses either the genetic or the image information (but not both), which highlights the importance of an integrative analysis. The benefits of incorporating structural information in the proposed approach are further highlighted when compared with an alternate imaging genetics based analysis that uses elastic net for model fitting, ignoring the spatial configuration of the voxels.

The rest of the article is structured as follows. Section 2 develops the methodology, details the prior specifications and potential hyperparameter choices and outlines the posterior computation steps. Section 3 provides the results from our analysis of ADNI dataset, while Section 4 provides additional discussions.

## 2. Materials and methods

### 2.1. Data sources and preprocessing steps

Data sources: This study utilizes data obtained from the Alzheimer's Disease Neuroimaging Initiative (ADNI), a project funded by the National Institutes of Health (NIH) and launched in 2004. ADNI's mission is to collect and share longitudinal data, including serial magnetic resonance imaging (MRI), positron emission tomography (PET), Mini-Mental State Examination (MMSE) scores, genetics information, other clinical or biological markers, and demographic data, to predict and prevent mild cognitive impairment (MCI) and early AD.

For this study, we utilized data from the ADNI 1 program collected at baseline, 6-month follow-up (M06), and 12-month follow-up (M12) intervals. The dataset consisted of MRI data, gene expression data obtained from blood samples, basic demographic data including gender, age, and APOE, as well as MMSE scores. With this comprehensive dataset, our objective was to predict the MMSE score based on voxel-level cortical thickness measurements derived from T1w-MRI imaging data and mRNA gene expression data. Our analysis accounts for the spatial configurations of the voxels as well as dependency between genes, in the regression model.

Demographic and cognitive data description: In this study, we analyzed a dataset consisting of 119 subjects with MCI from ADNI-1, for whom both imaging and transcriptomics data were available. The subjects' APOE status, gender, and age remained consistent across the three time points of the study: baseline, month 6 (M06), and month 12 (M12). Of the 119 subjects, 33 (27.7%) were female and 86 (72.3%) were male, with a mean age of 74.0 years and a standard deviation of 6.59. The majority of subjects had an APOE value of 0 (48.7%), followed by 1 (39.5%) and 2 (11.8%). The cognitive measurements for subjects were recorded in terms of Mini-Mental State Examination (MMSE) scores at baseline, M06, and M12. At baseline, the MMSE scores had a mean of 27.4 and a standard deviation of 1.72. At M06, the MMSE scores had a mean of 27.0 and a standard deviation of 2.25, and at M12, the MMSE scores had a mean of 27.0 and a standard deviation of 2.53. A summary is provided in Table 1.

**Table 1:** Summary of demographic variables and cognitive measurements under study.

	**Overall**
	**(*N* = 118)**
**Age**	
Mean (SD)	74.00 (6.61)
Median [Min, Max]	73.75 [57.80, 86.70]
**Gender**	
Female	32 (27.1%)
Male	86 (72.9%)
**APOE4**	
0	58 (49.1%)
1	46 (39.0%)
2	14 (11.9%)
**Baseline MMSE Score**	
Mean (SD)	27.44 (1.70)
Median [Min, Max]	27.00 [24.00, 30.00]
**Month 6 MMSE Score**	
Mean (SD)	27.03 (2.26)
Median [Min, Max]	28.00 [21.00, 30.00]
**Month 12 MMSE Score**	
Mean (SD)	27.02 (2.54)
Median [Min, Max]	28.0 [17.00, 30.00]

Imaging data pre-processing: The T1-weighted MRI images were processed with the Advanced Normalization Tools (ANTs) registration pipeline (Tustison et al., 2014). All images were registered to a population-based template image to ensure that the brain locations from different participants were normalized to the same template space. The population-based template image was created based on 52 normal control participants from ADNI 1 and shared to us from the ANTs group (Tustison et al., 2019). Among other things, the ANTs pipeline (i) uses the N4 bias correction step to correct for intensity nonuniformity (Tustison et al., 2010), which inherently normalizes the intensity across samples; and (ii) implements a symmetric diffeomorphic image registration algorithm that performs spatial normalization (Avants et al., 2008), which aligns each participant's T1 images to a template brain image so that the images across different participants can be spatially comparable. Additionally, the processed brain images, estimated brain masks, and template tissue labels were used to run 6-tissue Atropos segmentation and generate tissue masks for cerebrospinal fluid (CSF), gray matter (GM), white matter (WM), deep gray matter (DGM), brain stem, and cerebellum. In this step, the tissue masks from the template image act as priors which inform the segmentation for each observation scan. Lastly, cortical thickness measurements were obtained using the DiReCT algorithm. The 3-D image was downsampled to dimension 48 × 48 × 48, and subsequently divided into 48 different 2-D sagittal slices to be used for analysis where each slice had dimension 48 × 48.

**Figure 1:**
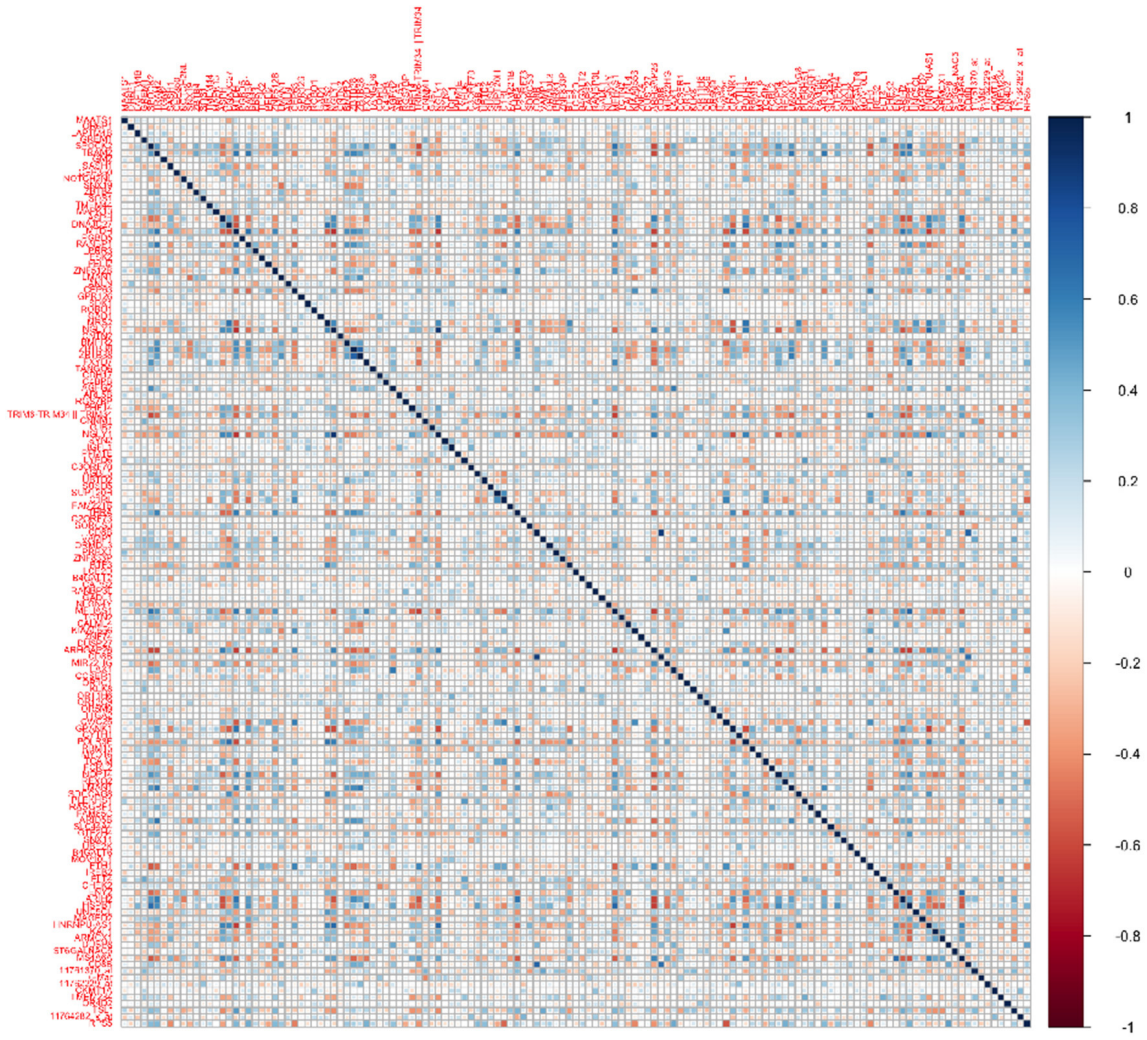
Heatmap for correlations between the 139 genes used for analysis.

Transcriptomics data: We performed a screening step to select a subset of the most promising genes for our analysis, from an overall 49,386 gene expression profiles in the ADNI data. We computed the correlations between the transcriptomics expressions for each gene and the cognitive scores at baseline, month 6, and month 12. Subsequently, we narrowed down the list of genes to those that exhibited significant correlations (at level 0.05) with the cognitive scores at all three visits. This screening strategy left us with a subset of 139 genes to be used for subsequent analysis. We also computed the correlations between this subset of transcriptomics features (Figure 1), which illustrates non-trivial correlations between several pairs of genes that need to be accounted for in our modeling framework.

### 2.2. Modeling framework

Let y∈Y denote a response variable that is regressed on scalar predictors $z\in X\subset R^{p}$ and $z_{1}\in X_{1}\subset R^{q}$ and a tensor predictor $\text{X}\in {⊗}_{j=1}^{D}R^{p_{j}}$. In terms of the motivating example, ***z*** may denote a set of demographic variables, ***z***_1_ may denote a set of genetic predictors and **X** may represent an image. We consider a scalar-on-image regression model that also incorporates the genetic and demographic predictors as given below:


(1) $$\begin{array}{l} y_{i}=\alpha +z_{i}{}^{\prime }\gamma +{\left(z_{1}\right)}_{i}^{\prime }\eta +\langle X_{i},B\rangle +\varepsilon_{i},i=1,\cdots \;,n \end{array}$$

where α is the intercept term, ***γ*** is a *p*×1 coefficient vector corresponding to the scalar predictors ***z***, ***η*** is a *q*×1 coefficient vector corresponding to the scalar predictors ***z***_1_, $\text{B}\in {⊗}_{j=1}^{D}R^{p_{j}}$ denotes the tensor parameter corresponding to the tensor predictor **X** and *n* denotes the total number of subjects in the study. We assume that the random error term ε_*i*_, *i* = 1, ⋯ , *n*, is normally distributed with mean 0 and variance σ^2^. Note that the coefficient tensor B has $\prod_{j=1}^{D}p_{j}$ elements which leads to severe parameter proliferation. To address this issue, we use a rank-R PARAFAC decomposition for **B** following the approach in Guhaniyogi et al. (2017), which leads to a significant parameter reduction. Thus we have,


$$\begin{array}{l} \text{B}=\overset{R}{\underset{r=1}{\sum }}\beta_{1}^{\left(r\right)}\circ \cdots \circ \beta_{D}^{\left(r\right)} \end{array}$$

where $\beta_{j}^{\left(r\right)},1\leq j\leq D$ and 1 ≤ *r* ≤ *R*, are the *p*_*j*_×1 dimensional tensor margins. Then the tensor inner product in (1) becomes $\langle {\text{X}}_{i},\overset{R}{\underset{r=1}{\sum }}\beta_{1}^{\left(r\right)}•\cdots •\beta_{D}^{\left(r\right)}\rangle =\underset{\left(i_{1},\cdots \;,i_{D}\right)}{\sum }{\left({\text{X}}_{i}\right)}_{i_{1},\cdots \;,i_{D}}{\left(\text{B}\right)}_{i_{1},\cdots \;,i_{D}}$ corresponding to the *i*th subject, where the parameter corresponding to voxel (*i*_1_, ⋯ , *i*_*D*_) of the image is given by:


(2) $$\begin{array}{l} {\left(\text{B}\right)}_{i_{1},\cdots \;,i_{D}}=\overset{R}{\underset{r=1}{\sum }}\overset{D}{\underset{j=1}{\prod }}\beta_{j,i_{j}}^{\left(r\right)},\;\left(i_{1},\cdots \;,i_{D}\right)\in V_{\text{B}}={⊗}_{j=1}^{D}\left\{1,\cdots \;,p_{j}\right\}. \end{array}$$

It can be easily seen that rank-R PARAFAC decomposition for **B** massively reduces the number of model parameters from $p+q+2+\prod_{j=1}^{D}p_{j}$ to $p+q+2+R\overset{D}{\underset{j=1}{\sum }}p_{j}$, which is critical for a scalable analysis involving high-dimensional features. The construction of the tensor coefficients via a PARAFAC representation also naturally accounts for spatial dependence between coefficients that is expected to address the issue of collinearity between the imaging features. Further, we will impose appropriate shrinkage priors on the tensor margins, that can adequately downweight the tensor coefficients corresponding to unimportant brain regions and allocate non-trivial tensor coefficient values corresponding to important signals. The ultimate goal is to perform accurate prediction under these models for a suitably chosen prior distribution on the tensor coefficient. We denote the proposed method as integrative Bayesian tensor regression or *iBTR*.

**Figure 2:**
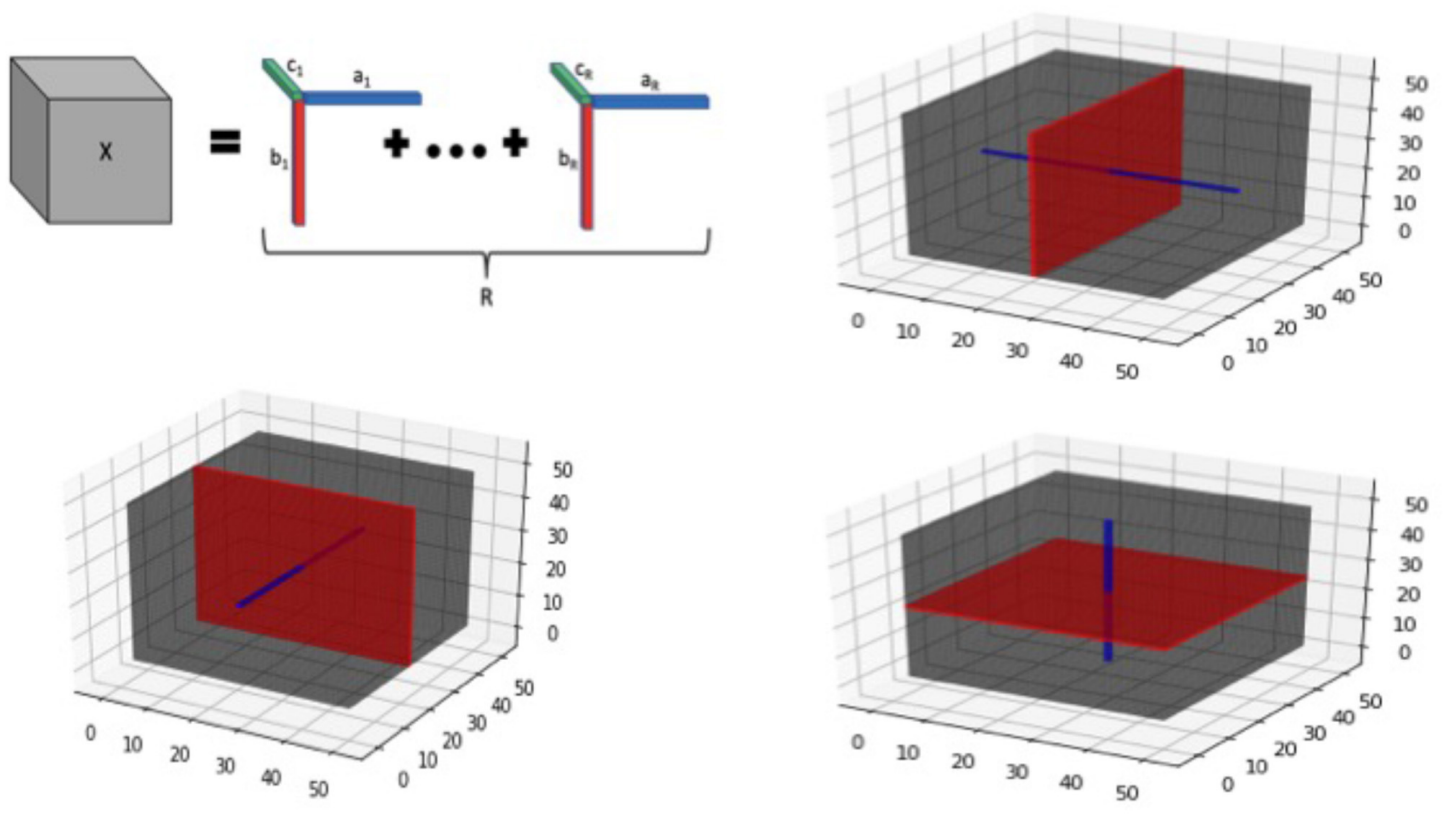
Tensor visualization for 3-dimensional image. **Top left panel** provides a graphic of a rank-*R* tensor decomposition for a 3-dimensional tensor *X*, represented as the sum of tensor products of vectors *a*_*r*_, *b*_*r*_, and *c*_*r*_, 1 ≤ *r* ≤ *R*. The **remaining panels** illustrate tensor slices (red) and fibers (blue) corresponding to each of the 3 dimensions of a 3-way tensor cube.

Additional details about tensor structure: We note that the tensor margins $\beta_{j,i_{j}}^{\left(r\right)}$ are only identifiable upto a permutation and multiplicative constant, unless some additional constraints are imposed. However, the lack of identifiability of tensor margins does not pose any issues for our setting, since the tensor product **B** is fully identifiable that is sufficient for our primary goal of coefficient estimation. Hence we do not impose any additional identifiability conditions on the tensor margins, which is consistent with Bayesian tensor modeling literature (Guhaniyogi et al., 2017). The tensor decomposition is visually illustrated in Figure 2 for the three-dimensional case.

In addition to the tensor margins, there are other lower-dimensional objects that are naturally embedded within a tensor structure. These include tensor fibers that can be visualized as a thin thread of points generated when varying only one of the tensor modes, while keeping the other modes fixed. For example for a three-way tensor (*D* = 3), mode-1 fibers correspond to the collection of *d*_1_-dimensional vectors that are obtained by fixing the tensor modes (axes) for modes 2 and 3, while varying the coordinates of the mode 1. Mode-2 and Mode-3 fibers can be defined similarly. On the other hand, tensor slices are defined as lower dimensional sub-spaces of a tensor that are generated by varying two tensor modes simultaneously, while keeping the third tensor mode fixed (for the *D* = 3 case). The tensor fibers and slices are illustrated in Figure 2 (all panels except the top left one), and these structures will be useful for understanding different aspects of the model in the sequel. For example, tensor fibers and slices will be directly instrumental for estimating the tensor margins in a robust manner, even in the presence of sparsity in the images as outlined in the sequel.

Preserving spatial configurations: Before fitting the tensor model, the voxels in the image are mapped to a regularly spaced grid that is more amenable to a tensor-based treatment. Such a mapping preserves the spatial configurations of the voxels that provides significant benefits over a univariate voxel-wise analysis or a multivariable analysis that vectorizes the voxels without regard for spatial configurations. While the grid mapping may not preserve the exact spatial distances between voxels, this is not a major concern in practice. This is because the tensor model can capture correlations between neighboring elements in the tensor margins, which allows for effective analysis of the spatial relationships between voxels. To better understand how spatial smoothing is induced between the regression coefficients for neighboring voxels in the 3-D case, note that the tensor coefficients for Γ corresponding to the neighboring voxels (*k*_1_, *k*_2_, *k*_3_) and $\left(k_{1}^{*},k_{2},k_{3}\right)$ for $k_{1}\neq k_{1}^{*}$ are given as $B_{\left(k_{1},k_{2},k_{3}\right)}=\overset{R}{\underset{r=1}{\sum }}\beta_{1k_{1}}^{\left(r\right)}\beta_{2k_{2}}^{\left(r\right)}\beta_{3k_{3}}^{\left(r\right)},$ and $B_{\left(k_{1}^{*},k_{2},k_{3}\right)}=\overset{R}{\underset{r=1}{\sum }}\beta_{1k_{1}^{*}}^{\left(r\right)}\beta_{2k_{2}}^{\left(r\right)}\beta_{3k_{3}}^{\left(r\right)},$ respectively. These coefficients share many common elements from the tensor margins $\beta_{2}^{\left(r\right)},\beta_{3}^{\left(r\right)}$ that induces spatial smoothing and therefore preserves spatial configuration.

Pooling information across voxels: A desirable feature of the tensor construction is that it is able to estimate voxel-specific coefficients using the information from neighboring voxels by estimating the tensor margins under the inherent low-rank structure. This feature yields more accurate results that are more robust to missing voxels and noise in the images and provides immediate advantages over univariate or multivariate voxel-wise methods that are not equipped to pool information across voxels. Consider the following 3-D toy illustration involving the estimation of the element ***B***_(1, 3, 1)_ that is expressed as $\overset{R}{\underset{r=1}{\sum }}\beta_{11}^{\left(r\right)}\beta_{23}^{\left(r\right)}\beta_{31}^{\left(r\right)}$. The estimation of coefficients proceeds through the estimation of the tensor margins $\left\{\left(\beta_{1}^{\left(r\right)},\beta_{2}^{\left(r\right)},\beta_{3}^{\left(r\right)}\right):r=1,\ldots ,R\right\}$. We note that the elements $\left\{\beta_{11}^{\left(r\right)},r=1,\ldots ,R\right\}$ are inherently contained in the tensor coefficients corresponding to all voxels in the tensor slice given by {(1, *k*_2_, *k*_3_), *k*_2_ = 1, …, *p*_2_, *k*_3_ = 1, …, *p*_3_} (refer to Figure 1). Similarly, the tensor margin elements $\left\{\beta_{23}^{\left(r\right)},r=1,\ldots ,R\right\}$ are contained in the tensor coefficients corresponding to the tensor slice {(*k*_1_, 3, *k*_3_), *k*_1_ = 1, …, *p*_1_, *k*_3_ = 1, …, *p*_3_}, a similar interpretation holds for the remaining tensor margin elements. Hence by pooling information across voxels contained in suitable tensor slices, the tensor margin parameters $\left\{\beta_{11}^{\left(r\right)},\beta_{23}^{\left(r\right)},\beta_{31}^{\left(r\right)},r=1,\ldots ,R\right\}$ can be learnt in a robust and effective manner. Similarly for the 2-D case, the tensor margin parameters are learnt by pooling information across tensor fibers.

Accommodating sparsity in images: Our analysis involves cortical thickness brain images that are inherently sparse due to the presence of many voxels in the brain that belong to other tissue types outside of the cortex. Further, due to brain atrophy in AD, one expects the presence of some voxels that have non-zero cortical thickness for only a subset of the samples. A desirable feature of the proposed model is that it is able to handle a small to moderate proportion of sparsity in the image. The proposed model uses tensor regression coefficients that are expressed as a low rank decomposition involving outer products of tensor margins. This allows for the estimation of voxel-specific coefficients corresponding to missing voxels in the image by pooling information across corresponding subsets of tensor slices (for 3-D image) and tensor fibers (for 2-D image) that comprise non-zero voxels. However, there is some loss of information due to missingness, but this loss is manageable when the proportion of zero voxels is not overly large. Furthermore, the model excludes voxels that have zero values across all samples, which may correspond to brain areas that do not belong to the brain cortex. Such voxels are screened out from analysis in our implementation. Overall, the proposed model seems to be designed to handle the sparsity and missingness of cortical thickness measurements in a reasonable manner, while still producing accurate results.

Dependency between transcriptomics features: The success of such a prediction model will also hinge heavily on the ability to account for collinearity between the transcriptomics predictors that is often exacerbated in high-dimensional settings and is expected to result in loss of prediction accuracy when not accounted for. Collinearity is not unexpected between transcriptomic features lying across multiple genes that often share some dependency since they lie on an underlying gene network or share common pathways. In our application, Figure 1 illustrates the correlations between transcriptomics factors that validate the need to address collinearity in the modeling framework. This will be addressed via a graph Laplacian prior on the genetic coefficients following the approach in Liu et al. (2014), which not only accounts for the unknown dependence structure across genes, but is also able to perform suitable regularization resulting in appropriate shrinkage.

### 2.3. Prior specifications

In this section, we first discuss the priors imposed on the key model parameters **B** and ***η*** in order to achieve parameter reduction. Following the approach in Guhaniyogi et al. (2017), we use the multiway Dirichlet generalized double Pareto (M-DGDP) prior for the tensor coefficient **B** and the hierarchical margin-level prior is given by


(3) $$\begin{array}{l} \beta_{j}^{\left(r\right)}\sim N(0,(\phi_{r}\tau )W_{jr}),w_{jr,k}\sim \text{Exp}(s_{jr}^{2}/2),s_{jr}\sim \text{Ga}(a_{\text{λ}},b_{\text{λ}}) \end{array}$$

As discussed in Guhaniyogi et al. (2017), this prior induces shrinkage across components in an exchangeable way. The global scale τ~Ga(*a*_τ_, *b*_τ_) has components τ_*r*_ = ϕ_*r*_τ, *r* = 1, ⋯ , *R* where Φ = (ϕ_1_, ⋯ , ϕ_*R*_)~Dirichlet(α_1_, ⋯ , α_*R*_) incorporates shrinkage toward lower ranks in the assumed PARAFAC decomposition. Also, **W**_*jr*_ = Diag(*w*_*jr*, 1_, ⋯ , *w*_*jr*,_*p*__*j*__), *j* = 1, ⋯ , *D, r* = 1, ⋯ , *R* represents margin-specific scale parameters for each component. Note that, $\beta_{j,k}^{\left(r\right)}|s_{jr},\phi_{r},\tau \overset{iid}{\sim }\text{DE}\left(s_{jr}/\sqrt{\phi_{r}\tau }\right),1\leq k\leq p_{j}$ , where DE refers to the Double Exponential distribution with the location parameter 0 and scale parameter $s_{jr}/\sqrt{\phi_{r}\tau }$. Thus the prior structure in (3) induces a GDP prior on the individual margin coefficients that has the form of an adaptive Lasso penalty (see Armagan et al., 2013). The use of element-specific scaling *w*_*jr, k*_ for modeling within-margin heterogeneity provides flexibility in estimation of ${\text{B}}_{r}=\left\{\beta_{j}^{\left(r\right)};1\leq j\leq D\right\}$. Common rate parameter *s*_*jr*_ incorporates shrinkage at the local scale by sharing information between margin elements. This prior also incorporates dimension reduction by favoring low-rank factorizations as discussed in Guhaniyogi et al. (2017).

Next, we use the Graph Laplacian prior (GL-prior) as outlined in Liu et al. (2014) for the coefficient vector ***η*** in order to incorporate the dependence structure through its precision matrix. Conditioning on σ^2^, the prior distribution for ***η*** takes the following form,


(4) $$\begin{array}{l} \eta |\sigma^{2}\sim N\left(0,\frac{\sigma^{2}}{m}{\text{Λ}}^{-1}\right) \end{array}$$

where the precision matrix ***Λ*** has the following structure


(5) $$\begin{array}{l} \Lambda =\\ \left[\begin{array}{cccc} 1+{\text{λ}}_{11}+\sum_{j\neq 1}\left|{\text{λ}}_{1j}\right| & {\text{λ}}_{12} & \ldots & {\text{λ}}_{1q}\\ {\text{λ}}_{21} & 1+{\text{λ}}_{22}+\sum_{j\neq 2}\left|{\text{λ}}_{2j}\right| & \ldots & {\text{λ}}_{2q}\\ \vdots & \vdots & \vdots & \vdots \\ {\text{λ}}_{q1} & \ldots & \ldots & 1+{\text{λ}}_{qq}+\sum_{j\neq q}\left|{\text{λ}}_{qj}\right| \end{array}\right] \end{array}$$

Where λ_*ij*_ = λ_*ji*_, λ_*ii*_>0 and the hyperparameter *m*≥0. Let ***λ*** denote the collection of all elements in ***Λ***. Then we propose the following prior distribution for ***λ***


(6) $$\begin{array}{l} \pi \left(\text{λ}\right)\propto C_{a,b}|\text{Λ}{|}^{-1/2}\overset{q}{\underset{i=1}{\prod }}{\text{λ}}_{ii}^{-3/2}\text{exp}\left(-\frac{a^{2}}{2{\text{λ}}_{ii}}\right)1\left({\text{λ}}_{ii}>0\right)\\ \;\underset{j<i}{\prod }|{\text{λ}}_{ij}{|}^{-3/2}\text{exp}\left(-\frac{b^{2}}{2|{\text{λ}}_{ij}|}\right) \end{array}$$

where *C*_*a,b*_ denotes the normalizing constant and *a,b* are the hyperparameters. Further we impose a conjugate inverse-gamma prior on the noise variance, $\sigma^{2}\sim \text{IG}\left(\nu /2,\nu s_{0}^{2}/2\right)$ with ν = 2 and $s_{0}^{2}$ is chosen by default so that *P*(σ^2^ ≤ 1) = 0.95 assuming a centered and scaled response. We specify a conjugate normal prior for the regression coefficients corresponding to demographic variables, $\gamma \sim N\left(0,\sigma^{2}\Sigma_{0\gamma }\right)$.

### 2.4. Selection of hyperparameters

Choice of hyperparameters of the Dirichlet component in multiway prior (3) plays an important role in controlling dimensionality of the model. Smaller values of hyperparameters leads to more component-specific scales τ_*r*_≈0, thus effectively collapsing on a low-rank tensor factorization (see Guhaniyogi et al., 2017 for more details). A discrete uniform prior is imposed on α over the default grid of 10 equally spaced values in [*R*^−*D*^, *R*^−0.10^]. The parameter α is then automatically tuned according to the degree of sparsity present. We impose discrete uniform prior on the hyperparameter *a*_λ_ over a default grid of 10 equally spaced values in [2, *D*+1] and then use $b_{\text{λ}}=\sqrt[{2D}]{a_{\text{λ}}}$ following the proposed choice in Guhaniyogi et al. (2017). For hyperparameters *m, a*, and *b* related to the GL-prior for ***η***, we consider the following prior:


(7) $$\begin{array}{l} \pi \left(m,a,b\right)\propto C_{a,b}^{-1}m^{h_{m}-1}\text{exp}\left(-h_{m}m\right)\text{exp}\left(-g_{a}a\right)\text{exp}\left(-g_{b}b\right) \end{array}$$

Small values of *g*_*a*_, *h*_*m*_, *g*_*b*_ are recommended to allow for a relatively flat prior. We set these values to 0.1 in our numerical experiments. Also, the results in the sequel show that (Tables 4–6) the prediction performance of our model is robust to choice of hyperparameters *g*_*a*_, *h*_*m*_ and *g*_*b*_.

Choice of tensor rank: To determine the optimal rank, we conducted a series of model runs with ranks ranging from 1 to 15, and chose the optimal tensor rank as that which minimizes a goodness of fit criteria called the Deviance Information Criterion (DIC) score. The DIC provides a measure of the quality of the fit of the model while accounting for its complexity by penalizing the incorporation of additional variables into the model (Li et al., 2017). Such an approach is consistent with other Bayesian tensor models routinely used in literature (Guhaniyogi et al., 2017). Although the rank selected using the DIC criteria may not always correspond to the rank that produces the lowest prediction error, but usually it is quite close to the best prediction across all the ranks considered in our experience.

### 2.5. Posterior computation

Having specified the priors for the model parameters, the next step is to obtain the joint posterior distribution of the model parameters which turns out to be intractable for closed-form computations. However, the full conditional posterior distributions of the model parameters are easy to sample from. We develop an efficient Gibbs sampling algorithm to sample from the full conditionals of the parameters which iterates through the following steps:

- Sample [α, Φ, τ|**B**, **W**] compositionally as [α|**B**, **W**][Φ, τ|α, **B**, **W**], following the steps as outlined in Guhaniyogi et al. (2017).
- Sample from $\left(\beta_{j}^{\left(r\right)},w_{jr},s_{jr},1\leq j\leq D,1\leq r\leq R\right)$ |Φ, τ, ***γ***, ***η***, σ, **y** using a back-fitting procedure to obtain a sequence of draws from the margin-level conditional distributions across components. Also, draw $\left[w_{jr},s_{jr}|\beta_{j}^{\left(r\right)},\phi_{r},\tau \right]=\left[w_{jr}|s_{jr},\beta_{j}^{\left(r\right)},\phi_{r},\tau \right]\left[s_{jr}|\beta_{j}^{\left(r\right)},\phi_{r},\tau \right]$.
  - Draw $s_{jr}\sim \text{Ga}\left(a_{\text{λ}}+p_{j},b_{\text{λ}}+{\beta_{j}^{\left(r\right)}}_{1}/\sqrt{\phi_{r}\tau }\right)$.
  - Draw $w_{jr,k}\sim \text{giG}\left(\frac{1}{2},s_{jr}^{2},\beta_{j,k}^{2\left(r\right)}/\left(\phi_{r}\tau \right)\right)$ independently for 1 ≤ *k* ≤ *p*_*j*_, where ‘giG' refers to generalized inverse Gaussian distribution, i.e. $X\sim f_{X}\left(x\right)=\text{giG}\left(p,a,b\right)\propto x^{p-1}exp\left(-\left(ax+b/x\right)/2\right)$.
  - Draw $\beta_{j}^{\left(r\right)}\sim N\left(\mu_{jr},\Sigma_{jr}\right)$ where $\mu_{jr}=\Sigma_{jr}H_{j}^{\left(r\right)}\tilde{y}/\sigma^{2}$,
    $\Sigma_{jr}={\left({H_{j}^{\left(r\right)}}^{\prime }H_{j}^{\left(r\right)}/\sigma^{2}+{{\text{W}}_{jr}}^{-1}/\left(\phi_{r}\tau \right)\right)}^{-1}$ and,

$$\begin{array}{lll} \;{\tilde{y}}_{i} & = & y_{i}-{z_{i}}^{\prime }\gamma -{{\left(z_{1}\right)}_{i}}^{\prime }\eta -\langle {\text{X}}_{i},\text{B}\rangle \\ H_{i,j}^{\left(r\right)} & = & {\left(h_{i,j,1}^{\left(r\right)},\cdots \;,h_{i,j,p_{j}}^{\left(r\right)}\right)}^{\prime }\text{ and,}\\ h_{i,j,k}^{\left(r\right)} & = & \overset{p_{1},\cdots \;,p_{D}}{\underset{d_{1}=1,\cdots \;,d_{D}=1}{\sum }}I\left(d_{j}=k\right)x_{d_{1},\cdots \;,d_{D}}\left(\underset{l\neq j}{\prod }\beta_{l,i_{l}}^{\left(r\right)}\right) \end{array}$$

- Sample ***γ***, σ|**B**, ***η***, **y** as follows:
  - Draw $\gamma \sim N\left(\mu_{\gamma },\sigma^{2}\Sigma_{\gamma }\right)$ where $\Sigma_{\gamma }={\left({\text{Z}}^{\prime }\text{Z}+\Sigma_{0,\gamma }^{-1}\right)}^{-1}$, $\mu_{\gamma }=\Sigma_{\gamma }{\text{Z}}^{\prime }{\text{y}}^{*}$ and ${\text{y}}_{i}^{*}={\text{y}}_{i}-{\left(z_{1}\right)}_{i}^{\prime }\eta -\langle {\text{X}}_{i},\text{B}\rangle$, $\text{Z}={\left(z_{1},\cdots \;,z_{n}\right)}^{\prime }$.
  - Draw $\sigma^{2}\sim \text{IG}\left(a_{\sigma },b_{\sigma }\right)$ where $a_{\sigma }=\left(n+\nu \right)/2,b_{\sigma }=\left(\nu s_{0}^{2}+{{\text{y}}^{*}}_{2}^{2}-{\text{y}}^{{*}^{\prime }}\text{Z}\mu_{\gamma }\right)/2$.

- Draw ***η*** from its full conditional: $\eta |\sigma^{2},\text{λ},\text{B},\text{X},\text{y}\sim N_{q}\left(\mu_{\eta },\Sigma_{\eta }\right)$ where,

$$\begin{array}{lll} \mu_{\eta } & = & {\left({\text{Z}}_{1}^{\prime }{\text{Z}}_{1}+m\Lambda \right)}^{-1}{\text{Z}}_{1}^{\prime }y^{**},\;\Sigma_{\eta }=\sigma^{2}{\left({\text{Z}}_{1}^{\prime }{\text{Z}}_{1}+m\Lambda \right)}^{-1}\text{and,}\\ y_{i}^{**} & = & y_{i}-{z_{i}}^{\prime }\gamma -\langle {\text{X}}_{i},\text{B}\rangle ,{\text{Z}}_{1}={\left({\left(z_{1}\right)}_{1},\cdots \;,{\left(z_{1}\right)}_{n}\right)}^{\prime }. \end{array}$$

- As the full conditional of ***λ*** does not have a closed form, we follow the same procedure outlined in Liu et al. (2014) for sampling of ***λ***.

### 2.6. Analysis plan

Our primary goal is to design an integrated strategy that combined spatially distributed voxel-level imaging features (downsampled), along with gene expression features and demographics data for predicting cognitive ability in MCI. We are particularly interested in concretely illustrating the benefits of embracing structural information in our integrative analysis, which include incorporating a tensor structure for the imaging voxel coefficients that preserve the spatial orientation of voxels, and simultaneously accounting for dependence between the transcriptomics features. Moreover, we perform the analysis at baseline and follow-up visits to illustrate the benefits of incorporating a structured brain imaging genetics analysis across time. As an additional but important analysis, we also analyse the ability of the proposed approach to predict the change in the cognitive scores across visits based on the change in the voxel-level cortical thickness differences between corresponding visits and transcriptomics factors as well as demographic features. Such an analysis will directly inform us about the ability of brain atrophy (structural changes) to predict the change in cognitive ability across time, which is of considerable clinical interest in AD research.

To demonstrate the performance of our novel approach, we tested several different alternative methods and compared their performance with the proposed method. At each visit, we implemented two tensor-based models that included (i) the proposed Bayesian tensor method with cortical thickness images and 139 gene expressions that is denoted as integrative Bayesian tensor regression or *iBTR*; and (ii) an imaging-only analysis that uses cortical thickness images for prediction using a Bayesian scalar-on-tensor regression that is denoted as Bayesian tensor regression or *BTR*. Additionally, we also compare the performance with (iii) an approach that vectorizes the imaging voxels and transcriptomics features and subsequently uses the elastic net for model fitting (denoted as *Elastic Net*); and (iv) a gene-only analysis that uses the transcriptomic measurements from 139 genes for prediction. We note that the elastic net is a combination of *L*_1_ and *L*_2_ penalties (Zou and Hastie, 2005), which results in sparse estimates and accounts for dependence within features. We note that demographic features such as age and gender were also included as additional covariates in each of the above predictive analysis.

The comparisons between (i) and (iv), and between (i) and (ii), highlight the necessity of incorporating imaging information along with transcriptomics features for obtaining superior prediction performance over an alternate analysis based on either an image-only or transcriptome-only analysis. In addition, the comparison with the Elastic Net approach (iii) that vectorizes the imaging voxels allows one to compare the benefits of preserving the spatial orientation of the voxels in predictive modeling under the proposed approach. In other words, this comparison allows us to investigate the loss in prediction accuracy when structural information in the images and the genes is not accounted for. We split the overall sample into 10 distinct training and test sets (80:20 ratio) and examined the out-of-sample prediction accuracy in terms of relative root mean squared error that is given as $RRMSE=\frac{\sum {\left(\hat{\theta_{i}}-\theta_{i}\right)}^{2}}{\frac{1}{n}\sum {\left(\theta_{i}-\bar{\theta }\right)}^{2}}$. Here $\theta ,\hat{\theta },$ correspond to the observed and estimated values, and $\bar{\theta }$ corresponds to the mean observed values. Clearly, a smaller relative RMSE value indicates superior prediction performance, while a higher value indicates poor performance and a value higher than one indicates that a performance that is even worse than a null model. In addition, we also examine convergence of the MCMC chains under the proposed method.

## 3. Results

**Figure 3:**
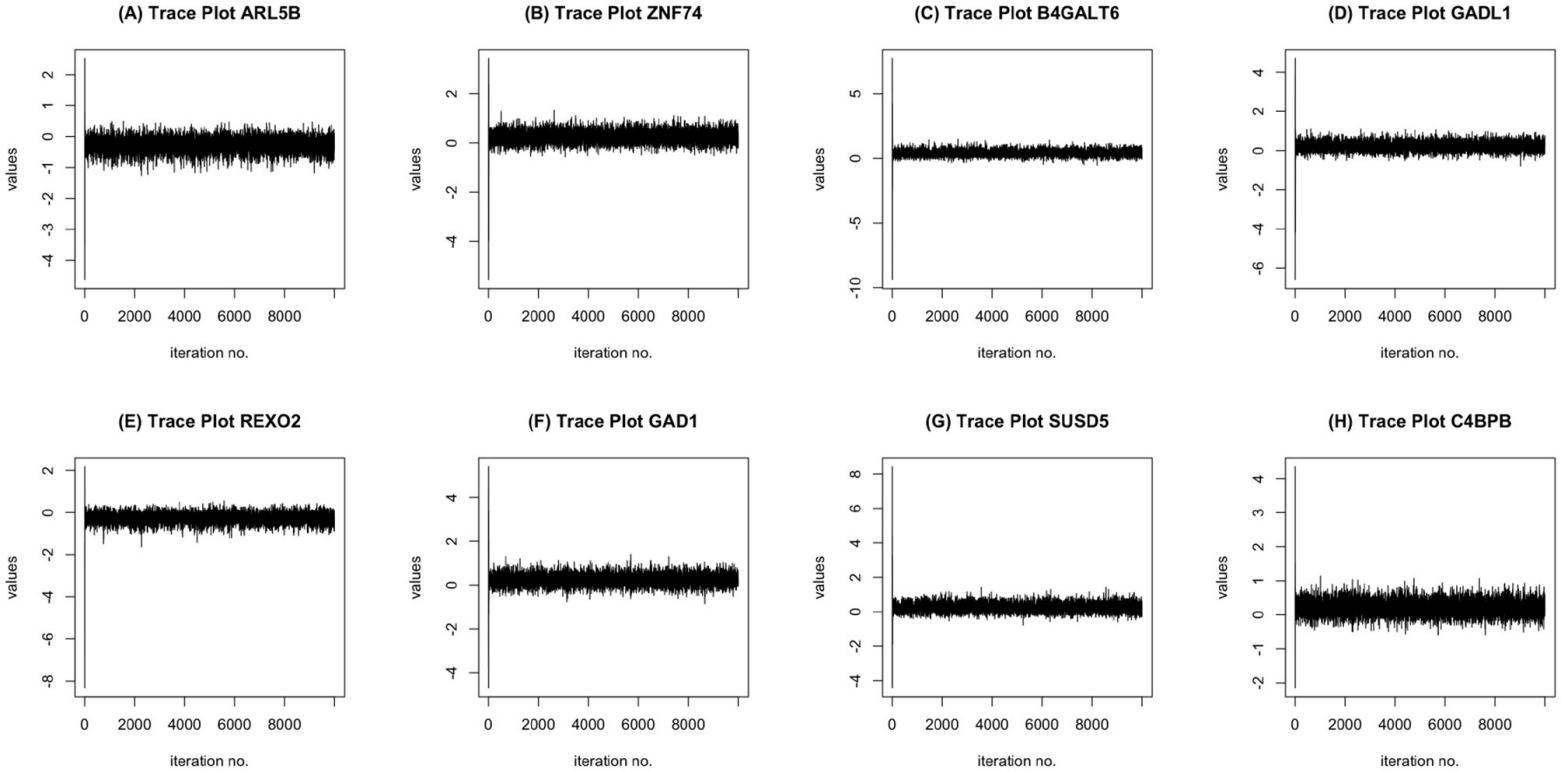
MCMC trace plots of some informative genes: ARL5B **(A)**, ZNF74 **(B)**, B4GALT6 **(C)**, GADL1 **(D)**, REXO2 **(E)**, GAD1 **(F)**, SUSD5 **(G)**, C4BPB **(H)**.

MCMC Convergence: We ran the MCMC chain for 10,000 iterations and tested for convergence for all covariates. Using the augmented Dickey-Fuller test, at 10,000 iterations and with a burn-in of 3,000 iterations, the MCMC chains for the coefficients corresponding to all of the voxels, transcriptomics factors, and demographic factors were stationary. To some degree, this indicates the suitability of the MCMC chain implemented in our approach. Some MCMC trace plots corresponding to the regression coefficients for a subset of genes in provided in Figure 3.

### 3.1. Choice of rank in tensor regression models

One of the key parameters of this model is the tensor rank, which plays a crucial role in determining the efficacy of the model when applied to different imaging and gene expression data as covariates. Moreover, the tensor ranks may potentially vary for each slice across the three longitudinal visits. Hence, it is imperative to choose the tensor ranks optimally.

**Figure 4:**
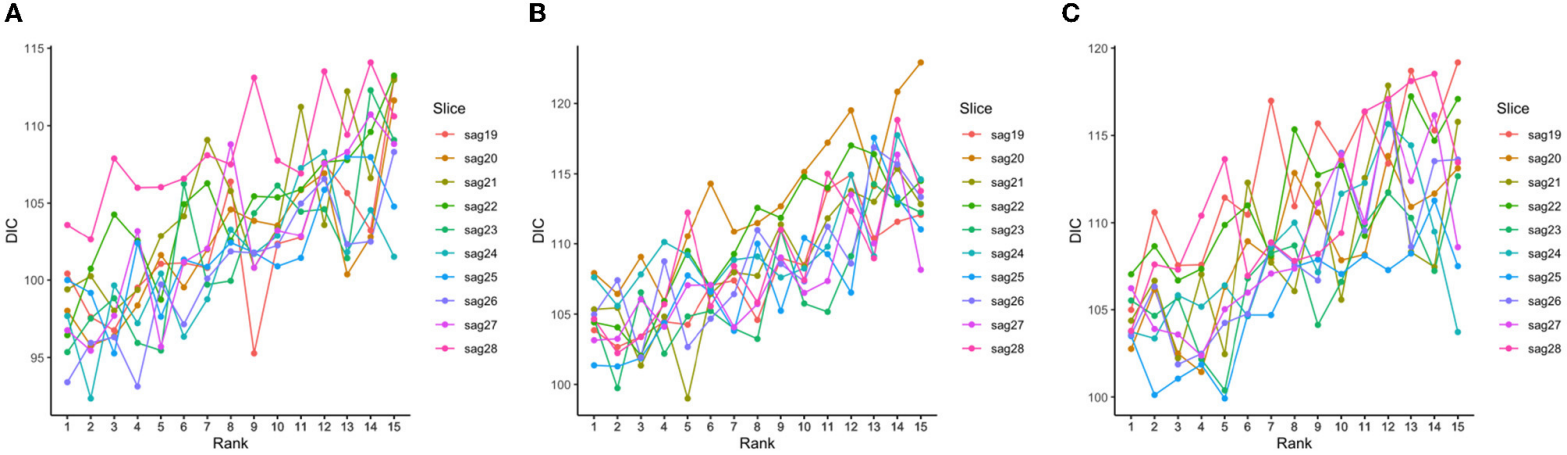
DIC values corresponding to different tensor ranks for the tensor model using both 139 genes and a subset of slices with less than 50% sparsity levels at baseline **(A)**, Month 6 **(B)**, and Month 12 **(C)**.

The tensor ranks chosen in our models and the corresponding DIC were listed in Table 2, and the trends of DIC by rank of our purposed models are shown in Figure 4.

**Table 2:** Reporting the optimal choice of rank for each slice.

	**Baseline**				**Month 06**				**Month 12**			
	**iBTR**		**BTR**		**iBTR**		**BTR**		**iBTR**		**BTR**	
Slice	Rank	DIC	Rank	DIC	Rank	DIC	Rank	DIC	Rank	DIC	Rank	DIC
19	9	95	3	127	2	103	4	133	1	105	1	147
20	2	96	2	129	4	106	6	146	4	101	2	143
21	3	98	3	129	5	99	2	141	3	102	3	146
22	1	96	3	131	3	102	3	138	3	107	1	148
23	1	95	3	132	2	100	1	135	5	100	5	138
24	2	92	2	131	2	106	1	150	2	103	3	136
25	3	95	3	131	2	101	3	138	5	100	1	135
26	4	93	2	128	3	102	3	141	3	102	2	139
27	2	95	6	151	1	103	1	141	4	102	4	138
28	2	103	1	148	2	102	2	142	1	104	1	147

We include only a subset of slices each having less than 50% non-zero voxels.

### 3.2. Prediction performance

Out of the 48 2-D slices considered (each of dimension 48 × 48), only a small number of slices contained non-zero cortical thickness measures for the majority of voxels in the slice (i.e. >50% of the voxels). We selected slices 19–28 for our analysis, each of which had at least 50 % voxels with non-zero cortical thickness. The remaining slices were excluded due to limited cortical thickness information and predominantly sparse patterns within each slice.

Cross-sectional results: Table 3 shows that our proposed Bayesian tensor method, which modeled the effects of 2-D cortical thickness image slices via a tensor decomposition coupled with transcriptomics factors, produced significantly lower relative RMSEs compared to the imaging-only analysis using a Bayesian scalar-on-tensor regression. Moreover, the proposed approach demonstrated significant improvements for all slices considered, when compared to prediction based on vectorized imaging and gene expressions under an elastic net that ignores the spatial configurations between the imaging voxel predictors. In addition, the prediction based on only the 139 gene expressions under an elastic net model produced inferior results, with relative RMSE of 0.874, 0.854, and 0.884 for baseline, M06, and M12 respectively. Finally, while the predictive performance varies slightly across the different 2-D slices, the proposed integrative Bayesian tensor regression consistently has a superior predictive performance compared to the competing methods across all the 10 slices considered. These results illustrate the substantial benefits of combining imaging and transcriptomics information when predicting cognitive scores across multiple longitudinal visits, while also simultaneously accounting for the underlying spatial structure of the image and inherent dependencies between genes. We also examined the sensitivity of the *iBTR* model to the choice of hyperparameters *g*_*a*_, *h*_*m*_ and *g*_*b*_. To that end, we used data from slice 20 of the brain image corresponding to month 6, along with 139 genes, and other clinical covariates and obtained the prediction performance of the *iBTR* model. From Tables 4–6, it is evident that the prediction performance remains largely unaffected (in fact there are negligible changes) when we vary these hyperparameters and it still remains superior to the competing approaches considered in Section 2.6. This illustrates the robustness of the proposed *iBTR* method in terms of the hyperparameters.

**Table 3:** Prediction performance (relative RMSE) values for modeling cognitive scores (MMSE) for baseline and two longitudinal visits.

	**Baseline**				**Month 6**				**Month 12**			
**Slice #**	**Sparsity**	**iBTR**	**EN**	**BTR**	**Sparsity**	**iBTR**	**EN**	**BTR**	**Sparsity**	**iBTR**	**EN**	**BTR**
19	0.47	0.793*	0.891	1.005	0.50	0.755*	0.926	1.035	0.49	0.788*	0.891	1.000
20	0.44	0.798*	0.947	1.002	0.46	0.746*	0.948	1.019	0.46	0.794*	0.962	1.001
21	0.43	0.789*	0.944	1.004	0.44	0.750*	0.961	1.029	0.43	0.797*	0.974	0.999
22	0.42	0.791*	0.963	1.000	0.43	0.751*	0.900	1.023	0.43	0.796*	0.959	1.001
23	0.43	0.795*	0.928	1.000	0.43	0.755*	1.003	1.035	0.43	0.802*	1.017	1.018
24	0.45	0.793*	0.990	1.000	0.45	0.750*	0.969	1.008	0.46	0.796*	0.913	1.005
25	0.46	0.796*	0.982	0.998	0.47	0.752*	0.936	1.026	0.47	0.801*	1.012	1.007
26	0.45	0.793*	0.980	1.003	0.45	0.748*	0.918	1.014	0.46	0.795*	0.983	0.996
27	0.45	0.790*	0.959	0.979	0.46	0.750*	0.899	1.007	0.47	0.795*	0.918	0.997
28	0.47	0.787*	0.944	0.976	0.46	0.753*	0.856	1.019	0.48	0.790*	0.905	0.977

Only a subset of slices having less than 50% non-zero voxels were included for analysis. EN refers to the elastic net model that used vectorized imaging and transcriptome features as predictors. An asterix (*) indicates significantly improved predictive performance compared to the other methods under a one-tailed t-test.

**Table 4:** Prediction performance (relative RMSE) values of iBTR model for varying choices of hyperparameters *g*_*a*_ and *h*_*m*_, keeping *g*_*b*_ fixed at 0.1.

***g*_*a*_\*h*_*m*_**	**0.5**	**1**	**3**	**5**
0.5	0.745	0.757	0.757	0.758
1	0.758	0.757	0.757	0.751
3	0.759	0.756	0.757	0.757
5	0.745	0.756	0.757	0.744

**Table 5:** Prediction performance (relative RMSE) values of iBTR model for varying choices of hyperparameters *g*_*b*_ and *h*_*m*_, keeping *g*_*a*_ fixed at 0.1.

***g*_*b*_\*h*_*m*_**	**0.5**	**1**	**3**	**5**
0.5	0.745	0.757	0.744	0.756
2	0.744	0.757	0.757	0.744
3	0.756	0.744	0.743	0.757
4	0.757	0.756	0.757	0.744

**Table 6:** Prediction performance (relative RMSE) values of iBTR model for varying choices of hyperparameters *g*_*a*_ and *g*_*b*_, keeping *h*_*m*_ fixed at 0.1.

***g*_*a*_\*g*_*b*_**	**0.5**	**1**	**3**	**5**
0.5	0.745	0.748	0.746	0.745
1	0.746	0.749	0.746	0.743
3	0.746	0.745	0.748	0.745
5	0.747	0.744	0.743	0.749

Longitudinal cognitive change score analysis: For this analysis, the goal is to predict the change in the cognitive scores between month 12 and baseline, based on the differences in the cortical thickness between visits, coupled with transcriptomics factors. The prediction results reported in Table 7 show that our proposed integrative Bayesian tensor method results in significant improvements in prediction performance across several slices, compared to the other competing approaches. In particular, the predictive accuracy under the proposed approach is significantly improved corresponding to slices 19, 20, 23, 25, 26, 27, and 28. The prediction accuracy is also higher (but not significantly better) for the remaining slices when compared to the other competing methods. In particular, all the competing methods reported relative RMSE above 1 in Table 7 for almost all cases, which is indicative of poor performance. Additionally, the predictive performance based on only the 139 transcriptomics features was also not desirable (relative RMSE of 1.009). These results indicate that superior ability of the proposed integrative Bayesian tensor method to predict cognitive changes across longitudinal visits. We note that the predictive performance varies with the different 2-D slices in the brain that is expected given that one expects certain changes in the cortical thickness corresponding to certain brain regions to be predictive of change in cognition, and based on the fact that not all brain regions will undergo cortical changes within a follow-up period of one year. To this end, we also performed a separate analysis to predict the changes in cognitive scores between month 6 follow-up and baseline. For this case, none of the proposed approaches performed well, which is potentially due to the fact that the structural changes in the brain in a short follow-up period of 6 months is expected to be limited and may not be immediately predictive of cognitive changes during this period. Our findings suggest that incorporating certain brain slices and gene expressions can significantly enhance the accuracy of predicting longitudinal cognitive changes, and therefore provide valuable insights for developing effective prediction models for cognitive impairment.

**Table 7:** Prediction performance for modeling longitudinal changes in cognitive scores based on differences in cortical thickness maps across visits and transcriptomics features.

	**Month 06-baseline**				**Month 12-baseline**			
**Slice #**	**Sparsity %**	**iBTR**	**Elastic net**	**BTR**	**Sparsity %**	**iBTR**	**Elastic net**	**BTR**
19	0.45	1.005	1.010	1.032	0.45	0.981*	1.036	1.009
20	0.43	1.003	1.022	1.027	0.42	0.978*	1.024	1.002
21	0.41	0.999	1.001	1.033	0.41	0.978	1.588	1.007
22	0.40	1.007	1.010	1.034	0.40	0.979	0.998	1.009
23	0.41	0.996*	1.050	1.023	0.41	0.973*	1.011	1.013
24	0.43	1.002	1.053	1.068	0.43	0.978	3.110	1.002
25	0.44	1.003	1.160	1.027	0.44	0.980*	1.012	1.007
26	0.43	1.008	1.036	1.029	0.43	0.879*	1.153	1.008
27	0.43	1.000	1.032	1.026	0.44	0.973*	1.017	1.007
28	0.44	0.999	1.052	1.026	0.46	0.975*	1.025	1.005

Only a subset of slices having < 50% non-zero voxels were included for analysis. The prediction for changes between baseline and month 6 follow-up is poor for all approaches due to the limited structural brain changes and cognitive decline in a short period of 6 months and is provided as a comparison to the month12-baseline analysis. This latter analysis shows meaningful predictive gains under the proposed iBTR approach. The symbol * is used to mark the slices for which the predictive accuracy under the proposed approach is significantly improved compared to the other competing methods under a single-tailed t-test.

### 3.3. Informative genes

From our integrated analysis of imaging and transcriptomics data on 10 2-D image slices (slices with zero's less than 50% which are slice 19 to slice 28) and three time points, the expression levels of 8 genes were found to be significantly associated with the MMSE cognitive outcome across multiple slices and more than one longitudinal visit. Utilizing our proposed model that employs selected 10 slices with gene expression and demographic data from three distinct time points, we identified several significant genes by combining our findings across the three visits and based on credible intervals derived from the posterior distribution. For each gene, we computed the proportion of times (out of total of 30 models developed) where it was inferred as significant. The frequency and proportion of cases where these genes were identified as important was documented in Table 8. Literature search reveals that almost all the top genes are indeed functionally related to either brain function or some types of neurological or psychiatric disorders.

**Table 8:** Informative genes with frequencies that each gene was significantly associated with the MMSE scores among the subset of slices included in Table 3 with < 50% sparsity levels at three time points and the corresponding percentage.

**Gene**	**Frequency**	**Percentage (%)**
ARL5B	20	66.67
ZNF74	19	63.33
B4GALT6	14	46.67
GADL1	12	40.00
REXO2	10	33.33
GAD1	8	26.67
SUSD5	8	26.67
C4BPB	7	23.33

For example, the ARL5B gene that is the top-ranked gene in terms of importance in our analysis, found to be significant in about two thirds of all models, has been reported to be connected to suicide attempts in major depressive disorder (Mullins et al., 2019). ZNF74 gene, found to be significant in 64% of all models, has been implicated as a neurological blood protein biomarker (Hillary et al., 2019), and related to AD (Wang et al., 2020). B4GALT6 gene, found to be significant in half of the models, has been reported to be related to depression severity (Ye et al., 2022). GAD1 gene, found to be significant in 41% of all models, interestingly, has been found to be highly and exclusively expressed in brain according to Human Protein Atlas (HPA) (Lee et al., 2018). Additionally, the GAD1 gene has been reported in the literature to be related to multiple neurological traits and disorders including Cognitive performance, Cognitive performance (MTAG) and attention deficit hyperactivity disorder, autism spectrum disorder and intelligence (Rao et al., 2022).

These findings convincingly demonstrated that our model is capable of identifying biologically-relevant genes, that together with MR imaging features, that can robustly predict the human cognitive abilities measured using the MMSE scores. Furthermore, the fact that these subset of genes in Table 8 were identified as important across multiple visits, illustrates the reproducibility of these findings that is crucial in AD studies.

### 3.4. Computational time of the iBTR model

**Figure 5:**
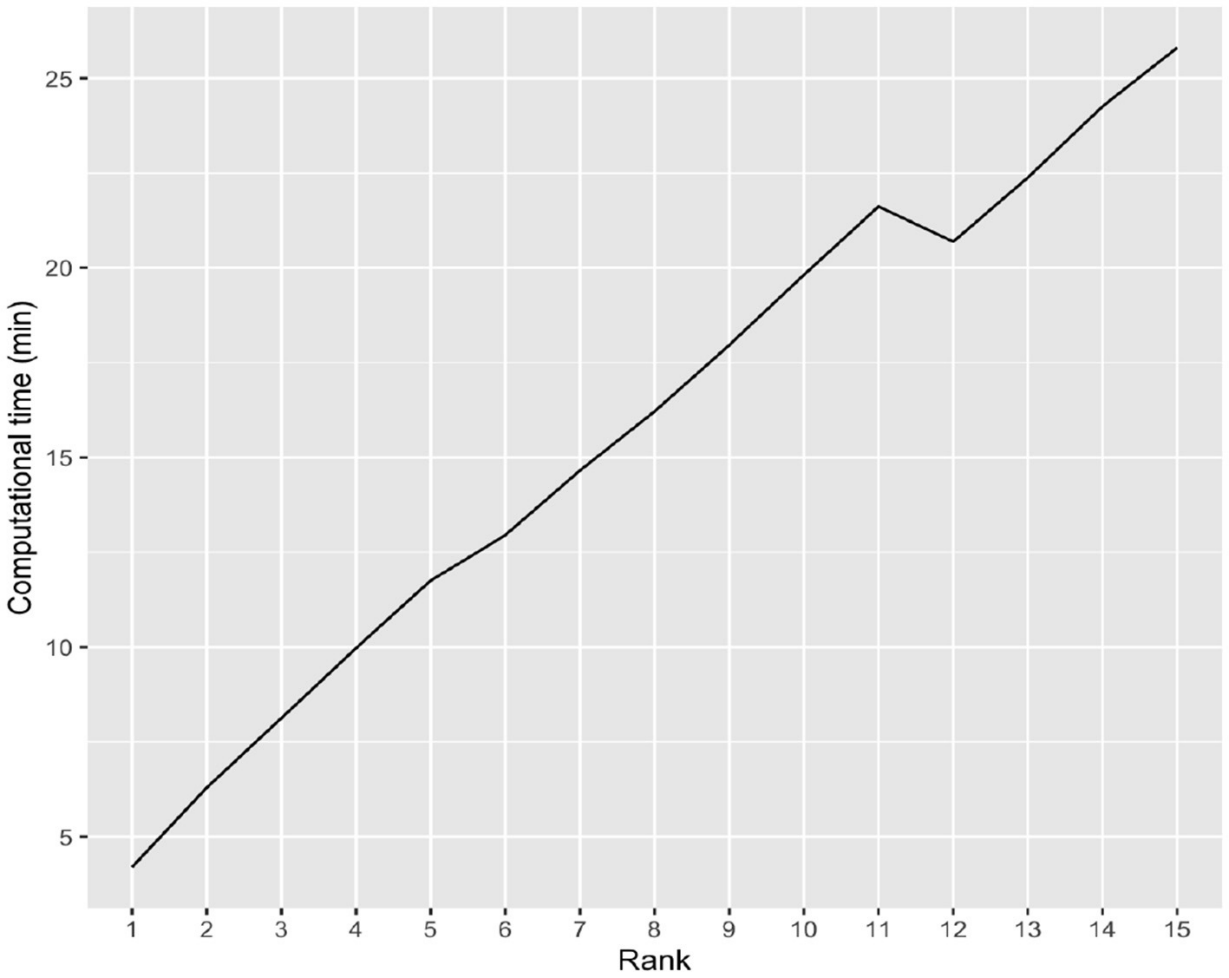
Computational time of the iBTR model with different ranks.

In high dimensional settings scalability or computational time taken by an algorithm is also very crucial. To that end, we examine the computational time of the *iBTR* model and run it with rank from 1 to 15 using data from slice 20 of the brain image corresponding to month 6, along with 139 genes, and other clinical covariates. The computational time taken by the iBTR model varies from 4 to 26 minutes for every 2,000 iterations, depending on the rank. The computational time is expected to increase as the tensor rank increases and/or the image size as well as the number of genetic covariates increase. Figure 5 illustrates the relationship between rank and computational time.

## 4. Discussion

AD is a chronic neurodegenerative disease that causes a slow but relentlessly progressive erosion of memory and cognition. It is the 6^*th*^ leading cause of death and 2^*nd*^ leading contributing cause of death (Heron et al., 2009). Unlike every other major cause of death, AD prevalence is rising (Heron et al., 2009), contributed by the rapid aging of the population and the lack of effective treatment options after disease onset. Therefore, identifying biomarkers that are predictive of AD progression, especially non-invasive ones well ahead of disease onset, is crucial in our effort of battling the scourge of AD.

Over the past decade, attempts have been made to identify imaging-based as well as genetics-based markers. Despite much progress, there is still much room for improvement in terms of finding the optimal types and combinations of imaging features and -omics modalities that are most predictive of cognitive decline or disease progression. Our study involving a novel integrative Bayesian model-based scalar-on-image regression approach that combines sparse cortical thickness imaging features with transcriptomics features to predict cognitive ability is expected to make a significant contribution in this regard. Our secondary analysis focused on modeling the change in cognitive outcomes illustrated the ability of cortical thickness changes in the brain to predict cognitive decline after accounting for transcriptomics factors. Although other methods have been developed that integrate imaging and genetics features, to the best of our knowledge, our method is the first that merge imaging and transcriptomics features under a tensor based model that accounts for the spatial configurations of the imaging voxels and underlying dependencies between genes. Compared to prediction using only imaging or only transcriptomics data, the results under our integrative model suggest that incorporating certain brain slices and gene expressions can significantly enhance the accuracy of predicting changes in MMSE scores. Moreover, incorporating the complex spatial organization in the image via a tensor-based approach as well as the dependence across transcriptomics features via structured priors provides conclusive prediction improvements over a naive analysis that vectorizes all imaging and -omics features to be used in the regression model.

Additionally, on top of the improved prediction model, the feature selection based on credible intervals under the Bayesian method can potentially provide a list of informed features, including genes, whose expression levels are shown to be informative of the cognitive ability of the patients. In particular, our cognitive prediction analysis for three longitudinal visits is able to detect important transcriptomics factors that are relevant across multiple visits and hence reproducible. Therefore, the corresponding genes can potentially provide promising therapeutic targets for downstream analysis. In-depth investigation of these informed genes indeed reveals that many of them have been reported to be related to neurological or psychiatric traits, hence it is made sense that their expression levels can contribute as potential biomarkers for the cognitive ability. Our work illustrates that it may be of interest to further explore transcriptomics features as potential biomarkers for AD, in combination with cortical thickness measurements.

There are multiple aspects where our model-based method can be further improved. For example, it may be helpful to incorporate informative priors for genes such as gene networks or underlying pathways based on annotations or historical data in the context of AD (Li et al., 2015). Other possible directions include incorporating multi-omics information, and using 3-D images instead of multiple 2-D slices. One can further investigate a battery of cognitive tests that go beyond the MMSE score investigated in our analysis. All of these issues can be potentially resolved under suitable generalizations of the proposed method, and will be explored in future work.

## Data availability statement

Publicly available datasets were analyzed in this study. This data can be found here: https://adni.loni.usc.edu.

## Ethics statement

We did secondary analysis of already collected and de-identified data that is publicly available. The requested details may be found in the ADNI data sharing website. The patients/participants provided their written informed consent to participate in this study.

## Author contributions

YL contributed in terms of performing the analysis and writing parts of the manuscript. NC contributed in terms of developing the MCMC code used for analysis and writing parts of the manuscript. ZQ contributed in terms of participating in designing the analysis plan and writing parts of the manuscript. SK contributed in terms of designing the analysis, writing and editing the manuscript, overseeing the project, and acquiring funding. All authors contributed to the article and approved the submitted version.

## References

[B1] Annese A. Manzari C. Lionetti C. Picardi E. Horner D. S. Chiara M.. (2018). Whole transcriptome profiling of late-onset alzheimer's disease patients provides insights into the molecular changes involved in the disease. Sci. Rep. 8, 4282. 10.1038/s41598-018-22701-2 29523845 PMC5844946

[B2] Armagan A. Dunson D. B. Lee J. (2013). Generalized double pareto shrinkage. Stat. Sin. 23, 119. 10.5705/ss.2011.048 PMC3903426 24478567

[B3] Association A. (2014). 2014 Alzheimer's disease facts and figures. Alzheimer's Dement. 10, e47–e92. 10.1016/j.jalz.2014.02.001 24818261

[B4] Avants B. B. Epstein C. L. Grossman M. Gee J. C. (2008). Symmetric diffeomorphic image registration with cross-correlation: evaluating automated labeling of elderly and neurodegenerative brain. Med. Image Anal. 12, 26–41. 10.1016/j.media.2007.06.004 17659998 PMC2276735

[B5] Bagyinszky E. Giau V. V. An S. A. (2020). Transcriptomics in alzheimer's disease: aspects and challenges. Int. J. Mol. Sci. 21:3517. 10.3390/ijms21103517 32429229 PMC7278930

[B6] Bellenguez C. Grenier-Boley B. Lambert J.-C. (2020). Genetics of Alzheimer's disease: where we are, and where we are going. Curr. Opin. Neurobiol. 61, 40–48. 10.1016/j.conb.2019.11.024 31863938

[B7] Bi X. Li L. Xu R. Xing Z. (2021). Pathogenic factors identification of brain imaging and gene in late mild cognitive impairment. Interdisciplinary Sci. 13, 511–520. 10.1007/s12539-021-00449-0 34106420

[B8] Brookmeyer R. Johnson E. Ziegler-Graham K. Arrighi H. M. (2007). Forecasting the global burden of alzheimer's disease. Alzheimer's Dement. 3, 186–191. 10.1016/j.jalz.2007.04.381 19595937

[B9] Chang C. Kundu S. Long Q. (2018). Scalable bayesian variable selection for structured high-dimensional data. Biometrics 74, 1372–1382. 10.1111/biom.12882 29738602 PMC6222001

[B10] Dartora C. M. de Moura L. V. Koole M. Marques da Silva A. M. (2022). Discriminating aging cognitive decline spectrum using pet and magnetic resonance image features. J. Alzheimer's Dis: 89, 977–991. 10.3233/JAD-215164 35988218

[B11] Du A.-T. Schuff N. Kramer J. H. Rosen H. J. Gorno-Tempini M. L. Rankin K.. (2007). Different regional patterns of cortical thinning in alzheimer's disease and frontotemporal dementia. Brain 130, 1159–1166. 10.1093/brain/awm016 17353226 PMC1853284

[B12] Dukart J. Sambataro F. Bertolino A. (2016). Accurate prediction of conversion to alzheimer's disease using imaging, genetic, and neuropsychological biomarkers. Journal of Alzheimer's Disease 49, 1143–1159. 10.3233/JAD-150570 26599054

[B13] Fjell A. M. Grydeland H. Krogsrud S. K. Amlien I. Rohani D. A. Ferschmann L.. (2015). Development and aging of cortical thickness correspond to genetic organization patterns. Proc. Nat. Acad. Sci. 112, 15462–15467. 10.1073/pnas.1508831112 26575625 PMC4687601

[B14] Frisoni G. B. Fox N. C. Jack Jr C. R. Scheltens P. Thompson P. M. (2010). The clinical use of structural mri in alzheimer disease. Nat. Rev. Neurol. 6, 67–77. 10.1038/nrneurol.2009.215 20139996 PMC2938772

[B15] Guhaniyogi R. Qamar S. Dunson D. B. (2017). Bayesian tensor regression. The J. Mach. Learn. Res. 18, 2733–2763.

[B16] Hao X. Bao Y. Guo Y. Yu M. Zhang D. Risacher S. L.. (2020). Multi-modal neuroimaging feature selection with consistent metric constraint for diagnosis of alzheimer's disease. Med. Image Anal. 60, 101625. 10.1016/j.media.2019.101625 31841947 PMC6980345

[B17] Harrison T. M. Mahmood Z. Lau E. P. Karacozoff A. M. Burggren A. C. Small G. W.. (2016). An Alzheimer's disease genetic risk score predicts longitudinal thinning of hippocampal complex subregions in healthy older adults. Eneuro. 3, ENEURO.0098-16.2016. 10.1523/ENEURO.0098-16.2016 27482534 PMC4945997

[B18] Heron M. Hoyert D. L. Murphy S. L. Xu J. Kochanek K. D. Tejada-Vera B. (2009). National vital statistics reports. National Vital Statistics Rep. 57, 14. 19788058

[B19] Hillary R. F. McCartney D. L. Harris S. E. Stevenson A. J. Seeboth A. Zhang Q.. (2019). Genome and epigenome wide studies of neurological protein biomarkers in the lothian birth cohort 1936. Nat. Commun. 10, 3160. 10.1038/s41467-019-11177-x 31320639 PMC6639385

[B20] Hofer E. Roshchupkin G. V. Adams H. H. Knol M. J. Lin H. Li S.. (2020). Genetic correlations and genome-wide associations of cortical structure in general population samples of 22,824 adults. Nat. Commun. 11, 4796. 10.1038/s41467-020-18367-y 32963231 PMC7508833

[B21] Kim B.-H. Choi Y.-H. Yang J.-J. Kim S. Nho K. Lee J.-M.. (2020). Identification of novel genes associated with cortical thickness in alzheimer's disease: systems biology approach to neuroimaging endophenotype. J. Alzheimer's Dis. 75, 531–545. 10.3233/JAD-191175 32310165

[B22] Kong D. An B. Zhang J. Zhu H. (2020). L2rm: low-rank linear regression models for high-dimensional matrix responses. J. Am. Stat. Assoc. 115, 403–424. 10.1080/01621459.2018.1555092 33408427 PMC7781207

[B23] Kong D. Giovanello K. S. Wang Y. Lin W. Lee E. Fan Y.. (2015). Predicting alzheimer's disease using combined imaging-whole genome snp data. J. Alzheimer's Dis. 46, 695–702. 10.3233/JAD-150164 25869783 PMC4583331

[B24] Lee J. J. Wedow R. Okbay A. Kong E. Maghzian O. Zacher M.. (2018). Gene discovery and polygenic prediction from a genome-wide association study of educational attainment in 1.1 million individuals. Nat. Genet. 50, 1112–1121. 10.1038/s41588-018-0147-3 30038396 PMC6393768

[B25] Li B. Sun Z. He Q. Zhu Y. Qin Z. (2015). Bayesian inference with historical data-based informative priors improves detection of differentially expressed genes. Bioinformatics 32, 682–689. 10.1093/bioinformatics/btv631 26519502 PMC4907396

[B26] Li L. Yu X. Sheng C. Jiang X. Zhang Q. Han Y.. (2022). A review of brain imaging biomarker genomics in alzheimer's disease: implementation and perspectives. Transl. Neurodegener. 11, 42. 10.1186/s40035-022-00315-z 36109823 PMC9476275

[B27] Li Y. Jun Y. Zeng T. (2017). Deviance Information Criterion for Bayesian Model Selection: Justification and Variation.

[B28] Liu F. Chakraborty S. Li F. Liu Y. Lozano A. C. (2014). Bayesian regularization via graph Laplacian. Bayesian Anal. 9, 449–474. 10.1214/14-BA860

[B29] McGue M. Bouchard Jr T. J. Iacono W. G. Lykken D. T. (1993). Behavioral genetics of cognitive ability: a life-span perspective. Nat. Nurture Psychol. 1, 59–76. 10.1037/10131-003

[B30] Mullins N. Bigdeli T. B. Borglum A. D. Coleman J. R. Demontis D. Mehta D.. (2019). Gwas of suicide attempt in psychiatric disorders and association with major depression polygenic risk scores. Am. J. Psychiatry 176, 651–660. 31164008 10.1176/appi.ajp.2019.18080957 PMC6675659

[B31] Nathoo F. S. Kong L. Zhu H. Initiative A. D. N. (2019). A review of statistical methods in imaging genetics. Can. J. Statist. 47, 108–131. 10.1002/cjs.11487 31274952 PMC6605768

[B32] Plomin R. Spinath F. M. (2002). Genetics and general cognitive ability (g). Trends Cogn. Sci. 6, 169–176. 10.1016/S1364-6613(00)01853-2 11912040

[B33] Rao S. Baranova A. Yao Y. Wang J. Zhang F. (2022). Genetic relationships between attention-deficit/hyperactivity disorder, autism spectrum disorder, and intelligence. Neuropsychobiology 81, 484–496. 10.1159/000525411 35764056

[B34] Sabuncu M. R. Buckner R. L. Smoller J. W. Lee P. H. Fischl B. Sperling R. A.. (2012). The association between a polygenic alzheimer score and cortical thickness in clinically normal subjects. Cerebral Cortex 22, 2653–2661. 10.1093/cercor/bhr348 22169231 PMC3464416

[B35] Shen L. Thompson P. M. (2019). Brain imaging genomics: integrated analysis and machine learning. Proc. IEEE. 108, 125–162. 10.1109/JPROC.2019.2947272 31902950 PMC6941751

[B36] Sims R. Hill M. Williams J. (2020). The multiplex model of the genetics of alzheimer's disease. Nat. Neurosci. 23, 311–322. 10.1038/s41593-020-0599-5 32112059

[B37] Tong T. Gray K. Gao Q. Chen L. Rueckert D. Initiative A. D. N.. (2017). Multi-modal classification of alzheimer's disease using nonlinear graph fusion. Pattern Recognit. 63, 171–181. 10.1016/j.patcog.2016.10.009

[B38] Tustison N. J. Avants B. B. Cook P. A. Zheng Y. Egan A. Yushkevich P. A.. (2010). N4itk: improved n3 bias correction. IEEE Trans. Med. Imaging 29, 1310–1320. 10.1109/TMI.2010.2046908 20378467 PMC3071855

[B39] Tustison N. J. Cook P. A. Klein A. Song G. Das S. R. Duda J. T.. (2014). Large-scale evaluation of ants and freesurfer cortical thickness measurements. Neuroimage 99, 166–179. 10.1016/j.neuroimage.2014.05.044 24879923

[B40] Tustison N. J. Holbrook A. J. Avants B. B. Roberts J. M. Cook P. A. Reagh Z. M.. (2019). Longitudinal mapping of cortical thickness measurements: An alzheimer's disease neuroimaging initiative-based evaluation study. J. Alzheimer's Dis. 71, 165–183. 10.3233/JAD-190283 31356207 PMC10204115

[B41] Wang H. Yang J. Schneider J. A. De Jager P. L. Bennett D. A. Zhang H.-Y. (2020). Genome-wide interaction analysis of pathological hallmarks in alzheimer's disease. Neurobiol. Aging 93, 61–68. 10.1016/j.neurobiolaging.2020.04.025 32450446 PMC9795865

[B42] Weston P. S. Nicholas J. M. Lehmann M. Ryan N. S. Liang Y. Macpherson K.. (2016). Presymptomatic cortical thinning in familial alzheimer disease: a longitudinal mri study. Neurology 87, 2050–2057. 10.1212/WNL.0000000000003322 27733562 PMC5109950

[B43] Ye J. Cheng S. Chu X. Wen Y. Cheng B. Liu L.. (2022). Associations between electronic devices use and common mental traits: A gene-environment interaction model using the uk biobank data. Addict. Biol. 27, e13111. 10.1111/adb.13111 34877740

[B44] Yu D. Wang L. Kong D. Zhu H. (2022). Mapping the genetic-imaging-clinical pathway with applications to alzheimer's disease. J. Am. Stat. Assoc. 117, 1–30. 10.1080/01621459.2022.2087658 37009529 PMC10062702

[B45] Zhang D. Wang Y. Zhou L. Yuan H. Shen D. Initiative A. D. N.. (2011). Multimodal classification of alzheimer's disease and mild cognitive impairment. Neuroimage 55, 856–867. 10.1016/j.neuroimage.2011.01.008 21236349 PMC3057360

[B46] Zou H. Hastie T. (2005). Regularization and variable selection via the elastic net. J. Royal Stat. Soc. 67, 301–320. 10.1111/j.1467-9868.2005.00503.x

